# Mitochondrial mutations in protein coding genes of respiratory chain including complexes IV, V, and mt-tRNA genes are associated risk factors for congenital heart disease

**DOI:** 10.17179/excli2022-5298

**Published:** 2022-11-03

**Authors:** Mohammad Mehdi Heidari, Mehri Khatami, Akram Kamalipour, Mustafa Kalantari, Mahsa Movahed, Mohammad Hayet Emmamy, Mehdi Hadadzadeh, José Bragança, Mohsen Namnabat, Bahareh Mazrouei

**Affiliations:** 1 Department of Biology, Yazd University, Yazd, Iran; 2 Department of Cardiac Surgery, Afshar Hospital, Shahid Sadoughi University of Medical Sciences, Yazd, Iran; 3 Faculty of Medicine and Biomedical Sciences, Algarve Biomedical Center Research Institute, University of Algarve, Faro, Portugal

**Keywords:** congenital heart disease, mitochondrial genome, mutation, mt-tRNA, in-silico analysis

## Abstract

Most studies aiming at unraveling the molecular events associated with cardiac congenital heart disease (CHD) have focused on the effect of mutations occurring in the nuclear genome. In recent years, a significant role has been attributed to mitochondria for correct heart development and maturation of cardiomyocytes. Moreover, numerous heart defects have been associated with nucleotide variations occurring in the mitochondrial genome, affecting mitochondrial functions and cardiac energy metabolism, including genes encoding for subunits of respiratory chain complexes. Therefore, mutations in the mitochondrial genome may be a major cause of heart disease, including CHD, and their identification and characterization can shed light on pathological mechanisms occurring during heart development. Here, we have analyzed mitochondrial genetic variants in previously reported mutational genome hotspots and the flanking regions of *mt-ND1, mt-ND2, mt-COXI, mt-COXII, mt-ATPase8, mt-ATPase6, mt-COXIII,* and *mt-tRNAs* (*Ile, Gln, Met, Trp, Ala, Asn, Cys, Tyr, Ser, Asp, *and* Lys*) encoding genes by polymerase chain reaction-single stranded conformation polymorphism (PCR-SSCP) in 200 patients with CHD, undergoing cardiac surgery. A total of 23 mitochondrial variations (5 missense mutations, 8 synonymous variations, and 10 nucleotide changes in tRNA encoding genes) were identified and included 16 novel variants. Additionally, we showed that intracellular ATP was significantly reduced (*P*=0.002) in CHD patients compared with healthy controls, suggesting that the mutations have an impact on mitochondrial energy production. Functional and structural alterations caused by the mitochondrial nucleotide variations in the gene products were studied *in-silico* and predicted to convey a predisposing risk factor for CHD. Further studies are necessary to better understand the mechanisms by which the alterations identified in the present study contribute to the development of CHD in patients.

## List of abbreviations

ASD atrial septal defects

ATP adenosine triphosphate

CHD congenital heart disease

CI Conservation Index

COXI Cytochrome C oxidase subunit 1

ETC electron transfer chain

mtDNA mitochondrial DNA

OXPHOS oxidative phosphorylation

PCR polymerase chain reaction

PDA patent ductus arteriosus

ROS reactive oxygen species

SSCP single-strand conformational polymorphism

TOF tetralogy of Fallot

tRNA transfer RNA

VSD ventricular septal defect

## Introduction

To date, hundreds of different pathogenic mitochondrial genome (mtDNA) mutations have been described in humans, many of which are associated with heart defects, ranging from arrhythmias to very complex cardiovascular disorders (Bray and Ballinger, 2017[8]; Tafti et al., 2018[42]; Mazzaccara et al., 2021[32]; Wang et al., 2021[45]). This diversity and lack of a cardiac-specific phenotype in patients harboring mutated mtDNA are a challenge for cardiologists. Data analysis of publicly available databases by bioinformatics approaches indicates a high prevalence of cardiac disease in individuals with mitochondrial dysfunction. Deleterious mutations of mtDNA have drastic consequences on patients' cardiac phenotypes and affect significantly the survival rate and early mortality in large pediatric and adult cohorts with heart disorders, in comparison to patients without mitochondrial defects (Zhao et al., 2021[48]; Gomes et al., 2022[13]). The true prevalence of the mtDNA-related cardiovascular disease is unknown, although new evidence estimates the prevalence worldwide of mtDNA disorders and the frequency of cardiac involvement, to be at least 1 in 15,000 (Bates et al., 2012[6]; Villar et al., 2013[44]; Mazzaccara et al., 2021[32]).

The rarity of cases, restricted access to human heart tissue, and the lack of reliable animal models for the study of mitochondrial diseases have limited the understanding of pathogenetic mechanisms and molecular pathways that link mitochondrial genome defects to cardiac dysfunction. Poor correlation and unconfirmed associations of genotype-phenotype in patients are other important limitations. Nevertheless, experimental and clinical evidence suggests that oxidative phosphorylation dysfunctions, impairing the production of ATP, may have a detrimental effect on heart muscle activity (Doenst et al., 2013[11]; Zhou and Tian, 2018[49]). The human mitochondrial genome is a double-stranded circular molecule of 16,569-bp, containing 13 genes that encode oxidative phosphorylation (OXPHOS) subunits of electron transfer chain (ETC) complexes, along with 2 rRNA genes, 22 tRNA genes, and non-coding regions that have critical roles in replication and transcription. In humans, the ETC is composed of 5 complexes: i) NADH dehydrogenase (Complex I); ii) succinate dehydrogenase (Complex II); iii) cytochrome B (Complex III); iv) cytochrome C oxidase (Complex IV); and v) ATP synthase (complex V) (Habbane and Montoya, 2021[14]). Unlike the nuclear genome, the copy number of the mtDNA is highly variable in human cells and differs from tissue to tissue (Lopaschuk et al., 2021[30]). Although many studies have linked the nuclear genome to a variety of heart diseases, others have indicated that mtDNA may also have variants that increase the risk of cardiovascular diseases (Bates et al., 2012[6]; Abaci et al., 2015[1]). Of interest, we have demonstrated a higher incidence of cardiovascular diseases, such as CHD, in individuals with higher nucleotide variations in the mtDNA (Khatami et al., 2016[26]). Mitochondrial mutations are potential risk factors for CHD due to deleterious consequences, such as impairment of mitochondria energy metabolism and increased production of reactive oxygen species (ROS) (Poznyak and Ivanova, 2020[34]). CHD is the most frequent congenital anomaly reported among infants, estimated to occur annually in 5-20 in 1000 live births worldwide (Wu et al., 2020[47]). The most common types of CHD are ventricular septal defect (VSD), atrial septal defects (ASD), tetralogy of Fallot (TOF), and patent ductus arteriosus (PDA) (Hayes-Lattin and Salmi, 2020[15]). These abnormalities in cardiac morphogenesis may result from abnormal embryological processes, mostly happening in the first 8 weeks of pregnancy. On day 21 of gestation, two mesoderm-derived cardiogenic strands migrate and fuse to form a primitive linear and beating heart tube that develops between days 24 and 35 of gestation. These early embryonic and fetal developmental steps involve particularly complex structural and morphogenic changes, which are very sensitive to variations in genetic and environmental cues and are responsible for major heart defects (Kloesel et al., 2016[27]). Despite an extensive effort undertaken worldwide to understand heart development, the complete molecular etiology and mechanisms leading to CHD are still not fully understood. Part of CHD, which is a complex disorder, may be contributed to pathogenic mitochondrial mutations, generally located in tRNA and OXPHOS-related genes (Abaci et al., 2015[1]). In the present study, we have evaluated the pathogenicity of sequence variations occurring in the genes encoding for the complexes IV and V of OXPHOS subunits in mtDNA, by performing a systematic screening to evaluate mtDNA mutations in a cohort of Iranian patients with CHD.

## Material and Methods

### Patients 

In the present case-control study, a total of 200 unrelated CHD pediatric patients (110 males and 90 females) were evaluated by common clinical examinations and biochemical investigations, by experienced pediatric cardiologists in the Department of Cardiology at the Afshar hospital, Yazd, Iran. All patients were undergoing heart surgery and presented with a non-syndromic CHD. Syndromic CHD patients such as Noonan, DiGeorge, Holt-Oram, Marfan, Charge, and Alagille syndromes were excluded from this research (Table 1(Tab. 1)). The age range of patients was from a minimum of 20 days old to 10 years old (mean age: 4.5 ± 1.7). The control group was constituted of 160 healthy individuals matched in age- and sex-matched to CHD individuals (90 males and 70 females; mean age: 4.8 ± 1.9). Control individuals had no family history of cardiovascular diseases or mitochondrial disorders, and were tested for normal electrocardiogram and biochemical parameters. There were no significant differences in the main characteristics, such as sex and age between the control and patient groups (*P* = 0.65 and 0.77, respectively). Blood samples were collected from patients and healthy control individuals to harvest the mtDNA and identify nucleotide changes in genes encoding mitochondrial IV and V complexes and 11 mt-tRNA genes (Ile, Gln, Met, Trp, Ala, Asn, Cys, Tyr, Ser, Asp, and Lys tRNAs), and in their flanking regions. Our study was conducted respecting the ethical guidelines of the 1975 Declaration of Helsinki. All research protocols were approved by the Ethics Committee of Yazd University. After obtaining informed consent from all participating subjects or legal representatives, total DNA was extracted from peripheral blood samples for molecular screening, using a DNA extraction kit (Qiagen Co, Tehran, Iran). Specific oligonucleotide primers were designed and synthesized. The selected coding and flanking regions were amplified by polymerase chain reaction (PCR) and subjected to single-strand conformational polymorphism (SSCP). The abnormal conformers experienced direct DNA sequencing for the identification of mutation positions.

### Mitochondrial DNA analysis by PCR-SSCP and DNA Sequencing

Selected coding mtDNA sequences and flanking regions of mt-ND1, mt-ND2, mt-COXI, mt-COXII, mt-ATPase8, mt-ATPase6, mt-COXIII, and mt-tRNAs^Ile^, mt-tRNA^Gln^, mt-tRNA^Met^, mt-tRNA^Trp^, mt-tRNA^Ala^, mt-tRNA^Asn^, mt-tRNA^Cys^, mt-tRNA^Tyr^, mt-tRNA^Ser^, mt-tRNA^Asp^, and mt-tRNA^Lys^ genes (length about 5491 bp, 33.15 % of mtDNA) were amplified by polymerase chain reaction (PCR) in an automated thermal cycler (MWG-Biotech Primus). These coding regions of mtDNA were amplified with a total of twenty-four (12 pairs) PCR-specific primers to get appropriate amplicons for the evaluation of products by single-stranded conformation polymorphism (SSCP) (Table 2(Tab. 2)). Specific primers for the amplification of the regions of interest were designed using primer-premier 5 software, based on the complete genome of Homo sapiens mitochondrion of the NCBI site (https://www.ncbi.nlm.nih.gov). The quality of primers and their probable secondary structures were assessed with Gene Runner (http://www.generunner.net/) and the Oligo analyzer software.

PCR reactions were performed in a final volume of 25 µL containing 20-30 ng of total DNA as the template, 1.5 mM dNTPs mixture, 0.2 µmol/l of each primer, 1.5 mM MgCl_2_, 1U of *Taq* polymerase (Qiagen, Tehran, Iran), and 1X PCR buffer after initial denaturation for 5 minutes at 95 °C. Amplification conditions were as follows: 30 cycles of denaturation at 95 ºC for 30 sec, annealing of each fragment according (ranging from 52-65 ºC) for 30-40 sec, and extension at 72 ºC for 50 sec, with a final extension at 72 ºC for 5 min. 

For amplification of fragments containing tRNA genes, touch-down PCR programs were used based on the following procedure: initial denaturation at 95 °C for 4 min, continued by 5 cycles of 95 °C for 50 sec, 65 °C for 50 sec, extended at 72 °C for 50 sec and then the PCR reactions followed by 30 cycles of 95 °C for 30 sec, 57 to 63 °C for 30 sec (due to different fragments), 72 °C for 50 sec and finished with a final extension at 72 ºC for 5 min. Amplified products were confirmed for size by electrophoresis on 1.5 % agarose gel and stained with ethidium bromide.

For the screening of mutations, a Single-Stranded Conformation Polymorphism (SSCP) analysis was performed. Single-stranded PCR products (heat-denatured at 94 ºC for 5 min and chilled on ice for 4 min) were loaded onto a 6 % polyacrylamide/TBE 0.5X gel containing, and visualized with standard silver staining according to the standardized protocol (Saker, 1998[37]). Cases with abnormal SSCP conformers and band migration shifts were sent to the Macrogene Company (Korea) for automated DNA sequencing.

### Isolation of lymphocytes from peripheral blood

5 mL of patient blood was diluted at a ratio of 1:2 with Hank's solution (Vandidaz, Tehran, Iran) within the first hour following the sample collection, and gently placed on a chilled tube containing 5 ml of Ficoll (Aminsan, Tehran, Iran). The solutions were centrifuged at 1500×g for 25 minutes until the lymphocyte-containing layer was formed. The lymphocyte layer was diluted in 15 mL of Hank's solution and centrifuged at 500×g for 15 minutes. Next, the supernatant was removed, and the pellet was suspended in 5 mL of Hank's solution, before centrifugation at 250×g for 20 minutes. The supernatant was discarded and the pellet was suspended in RPMI 1640 medium (Sigma, Tehran, Iran).

### Measurements of intracellular ATP 

The cellular ATP rates were assayed in approximately 120 lymphocyte cells of CHD and healthy control blood samples, by bioluminescence measurement using the Cell Titer-Glo Luminescent Cell Viability Assay kit (Promega), as previously described (Heidari et al., 2009[17]). The standard ATP curve was obtained with serial dilutions of 4 mM ATP solution (at 0.25, 0.5, 1.0, 2.0 and 4.0). After calibration according to the ATP standard, the ATP content of the lymphocyte cells was determined with a Sirius tube luminometer, Berthold defection system (Germany).

### Bioinformatics analysis

Computer-based algorithms and integrated approaches were used to predict changes in the structure and function of proteins encoded in mitochondria, due to amino acid substitutions caused by the mutations identified in mtDNA. Molecular Evolutionary Genetics Analysis 6 (MEGA7: https://www.ncbi.nlm.nih.gov/pmc/) and ClustalX tool (http://www.ebi.ac.uk/tools/clustalw2) were used for online multiple amino acid sequence alignments and determination of the sequences homology and evolutionarily Conservation Index (CI) within and between different species (*Homo sapiens, Bos taurus, Mus musculus, Rattus norvegicus, Gallus gallus, Pongo pygmaeus, Gorilla gorilla, Xenopus laevis, Colobus guereza, Lemur catta, Pan paniscus, Cebus albifrons, Drosophila melanogaster, Strongylocentrotus droebachiensis, and Caenorhabditis elegans*). Evolutionary conservation analysis and identification of functional regions in proteins were achieved by the ConSurf server (https://consurf.tau.ac.il/). The CI was then calculated by comparing the human mtDNA variants with various species. Particularly, CI≥75 % was considered as having functional potential. 

The analysis of pathogenicity of the whole mtDNA was accomplished through the human mitochondrial genome database (Mitomap: http://www.mitomap.org/MITOMAP), mitochondrial tRNA Informatics Predictor (MitoTIP: https://www.mitomap.org/MITOMAP/MitoTipInfo), and Mitomaster (https://www.mitomap.org/foswiki/bin/view/MITOMASTER/WebHome) approaches, which summarize the polymorphisms and mutations in human mitochondrial DNA and evaluate the potential biological significance of each variant. Protein domains were assessed from UniProt (https://www.uniprot.org/) and InterPro databases (http://www.ebi.ac.uk/interpro/). Furthermore, sequence homology-based approaches include PolyPhen-2 (http://genetics.bwh.harvard.edu/pph2/), PROVEAN (https://www.jcvi.org/research/provean), and SIFT (https://sift.bii.a-star.edu.sg/) programs were used for prediction and annotation of the structural and functional impact of mitochondrial non-synonymous mutations and the potential deleterious effect of missense mutations on protein function. Furthermore, EXPASY (Expert Protein Analysis System: http://www.expasy.org) and Prot-Scale tool (http://web.expasy.org/protocol) were used for the prediction of protein hydrophobicity or hydrophilicity in normal and mutant states, PSIPRED (http://bionf.cs.ucl.ac.uk/psipred) and SOSUI (https://harrier.nagahama-i-bio.ac.jp/sosui/) tools for predicting the secondary structure of normal and mutated proteins, MAMIT-tRNA (http://mamit-trna.u-strasbg.fr) for getting information about mitochondrial tRNA gene sequences, the secondary structural features of mitochondrial tRNAs, and reported mutations/polymorphisms in these tRNAs, and Mfold (http://www.unafold.org/mfold/applications/rna-folding-form.php) for anticipating of RNA structure in normal and mutated sequences. 

We also used three-dimensional structural analysis to determine the functional importance and pathogenicity prediction of non-synonymous mutations in Complex IV and V mitochondrial proteins, because the three-dimensional structure of some human mitochondrial proteins such as cytochrome C oxidase I, II, III, ATP synthase 6, and ATP synthase 8 have not yet been defined. The homology-based structural prediction was performed using services such as the SWISS-MODEL server (https://swissmodel.expasy.org), I-TASSER (https://zhanglab.dcmb.med.umich.edu/I-TASSER/), and AlphaFold protein structure database (https://alphafold.ebi.ac.uk/) were used for modeling of human mtDNA proteins. Finally, protein PyMol software (https://www.pymol.org/), ClusPro 2.0: Protein-protein docking software, LigPlot+ v.2.2 (2D-ligand-protein interaction diagrams), and Dimplot programs (Laskowski and Swindells, 2011[28]) were used for visualization of the 3D and 2D structures and the identification of binding variations within the protein and with other complex proteins. In this manner, the number and length of bonds in normal and mutant proteins and the hydrogen bonds and hydrophobic interactions of proteins were investigated and the molecular interaction plot in mutated and normal proteins was generated using Dimplot in LIGPLOT software.

### Statistical analysis

Fisher's exact probability tests were used to the comparison of categorical variables and to examine the association between mitochondrial tRNA mutations and the risk of CHD. The GraphPad Prism software was used for statistical analysis and values of P<0.05 were regarded as statistically significant. 

## Results

### Genetic analysis

To identify point mutations/sequence variations in the mitochondrial DNA that may be potentially associated with CHD, we first performed mutation screening assays in the mitochondrial genome of cells isolated from sporadic pediatric patients with CHD of variable severity. PCR-SSCP and DNA sequencing analyses were performed for 18 mitochondrial genes (flanking regions of *mt-ND1, ND2, COXI, COXII, ATPase8, ATPase6, COXIII, and mt-tRNAs^Ile^, tRNA^Gln^, tRNA^Met^, tRNA^Trp^, tRNA^Ala^, tRNA^Asn^, tRNA^Cys^, tRNA^Tyr^, tRNA^Ser^, tRNA^Asp^, *and* tRNA^Lys^* genes) in a total of 200 CHD patients and 160 healthy controls. A total of 23 point mutations were identified in the mitochondrial genes of the CHD patients enrolled in our study (5 missense mutations, 8 synonymous polymorphisms, and 10 variants in tRNA genes) (Table 3(Tab. 3)). The comparison of the mutations identified in our study with mutations reported in the MITOMAP repository and other genomic databases revealed that most of the nucleotide changes harbored by the patients in our study (17 variations: 8 in tRNA genes and 9 in protein-coding genes) have not been reported and are novel variants.

The 23 variants identified included three novel point mutations in the flanking regions of the *mt-COXI* gene in sixteen (8 %) patients, two variations in the *mt-COXII* gene in two (1 %) patients previously reported in non-CHD contexts, and one novel nucleotide variation in the mt-ATPase8 gene in eight (4 %) patients. We also identified seven nucleotide changes in the mt-ATPase6 gene in twenty-three (11.5 %) patients, among which 4 are novel variants and 3 have been described in a non-CHD context. Additionally, two variations, of which one is novel, were identified in the mt-tRNAIle gene in seven (3.5 %) patients, as well as another nucleotide change in the mt-tRNATrp gene in one (0.50 %) patient, two novel nucleotide variations in mt-tRNAAla gene in five (2.5 %) patients, two novel nucleotide changes in mt-tRNAAsn gene in eight (4 %) patients, one novel variation in mt-tRNASer gene in three (1.5 %) patients, one novel nucleotide variation in mt-tRNAAsp gene in eight (4 %) patients, and one novel nucleotide change in mt-tRNALys gene in eighteen (9 %) patients.

### Pathogenic prediction analysis for non-synonymous variations

Among the variants present in the mitochondrial samples analyzed, 5 missense mutations (2 in *mt-COXI* and 3 in *mt-ATPase6* genes) were predicted to be pathogenic or probably pathogenic by Bioinformatics analysis (SIFT, Polyphen-2, MutPred2, PROVEAN, PANTER, PMut, I-Mutant. 2, and PredictSNP). These missense mutations are m.7398C>T (Pro499Sre) and m.7418C>G (Phe505Leu) in the *mt-COXI* gene, m.8860A>G (Thr112Ala), m.8878C>G (Arg118Gly), and m.9041A>G (His172Arg) in mt-ATPase6 gene. In addition, 16 point mutations (69.56 %) identified in our samples (2 in *mt-COXI* gene, 1 in *mt-COXII* gene, 6 in *mt-ATPase6* gene, and 7 in *mt-tRNA* genes) were in heteroplasmic state and 7 nucleotide variations (30.44 %) were in the homoplasmic state (Table 4(Tab. 4)). As expected, homoplasmic variants are more common in individuals, while heteroplasmic variants are more likely to be rare variants that were only found in a few patients. 

Of interest, most of the mitochondrial variations are positioned in highly conserved sites and are predicted to result in structural and functional changes that have the potential to disrupt normal mitochondrial energy biosynthesis. Importantly, heteroplasmic missense variants were not detected in the cohort of healthy control subjects, and they have not been reported as neutral polymorphisms. The Fisher test revealed significant differences between the patient and control groups either among protein-coding mutations (*P*=0.001) or mt-tRNA variations (*P*= 0.031), thus indicating their association with CHD. Moreover, the missense variations showed higher pathogenicity in heteroplasmic state scores than homoplasmic states, which are associated with more deleterious functional consequences of non-synonymous heteroplasmic variations. 

### Pathogenic prediction evaluations for mt-tRNA variations

**Figure 1:**
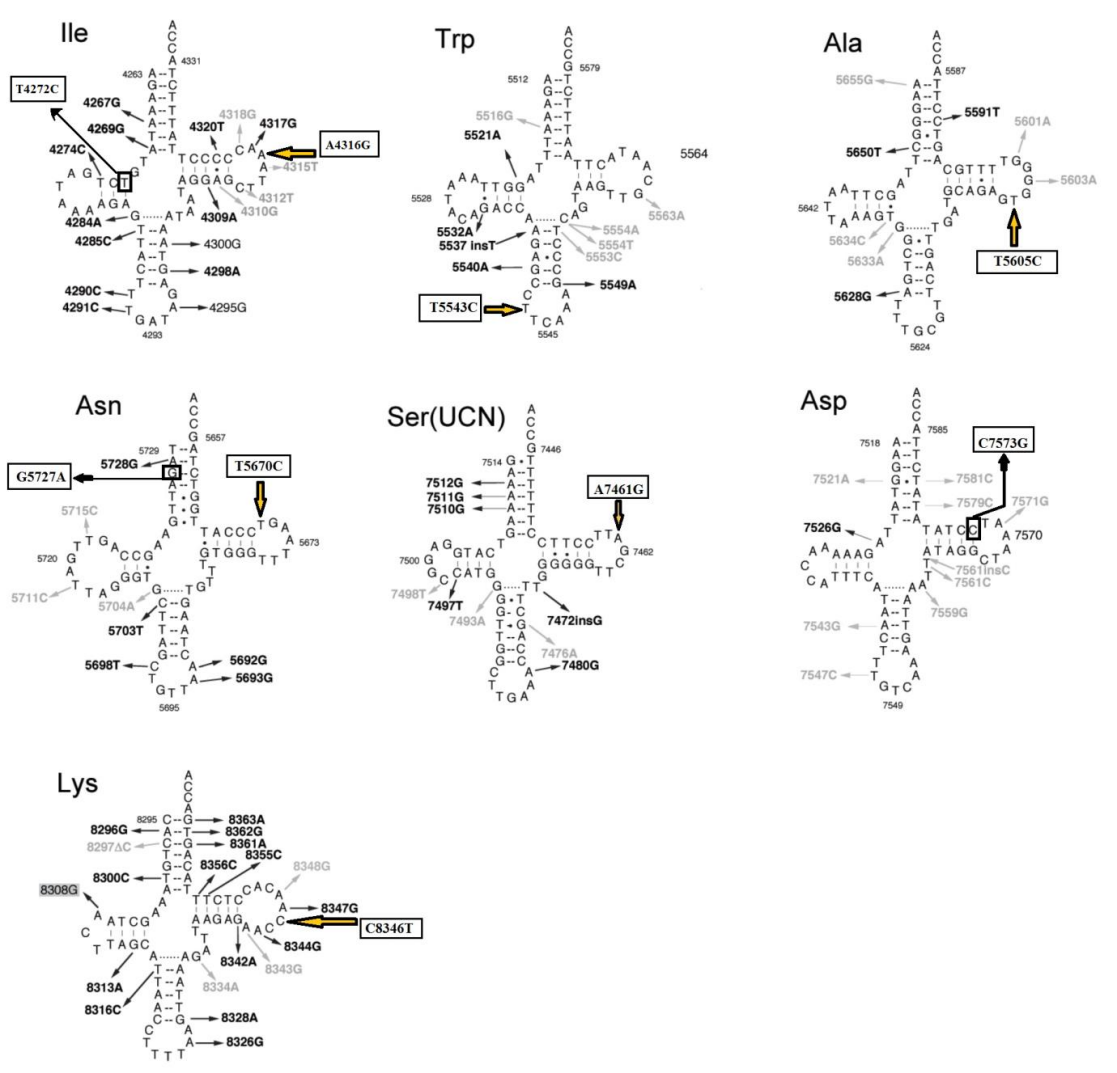
Position of nucleotide changes in the secondary structure of studied mt-tRNAs

Our results confirmed that eight *mt-tRNA* mutations may be pathogenic/likely pathogenic mutations and only two variants in tRNA genes (A43416G in *tRNA^Ile^* and C8346T in *tRNA^Lys^* genes) were likely benign (with pathogenicity scores: 37.10 % and 2.60 %, respectively) according to the MitoTIP predictor for tRNA variants (Sonney et al., 2017[41]). We also used the Mamit-tRNA tool for secondary structure prediction of mt-tRNAs and to define the position of each nucleotide change in tRNA structures, in a stem or a loop, or to assess whether the variants altered the classic Watson-Crick base-pairing (Figure 1(Fig. 1)). We observed that there was one variant occurring in the ACC-stem (G5727T in *tRNA^Asn^*), one variant in the DHU-stem (T4272C in *tRNA^Ile^*), one variant in TψC-stem (C7573G in *tRNA^Asp^*), five variants in TψC loop (A4316G in *tRNA^Ile^*, T5605C in *tRNA^Ala^*, T5670C in *tRNA^Asn^*, A7461G in *tRNA^Ser^*, and C8346T in *tRNA^Lys^*), one variant in anticodon loop (T5543C in *tRNA^Trp^*), and one variant in processing site of 5ʹ-terminus (A5656G in *tRNA^Ala^*). Of interest, 4 variants disrupted the classic Watson-Crick base pairings (those occurring at TψC loop, ACC-stem, and anticodon loop), whereas 2 variants created novel Watson-Crick base pairings (those occurring at DHU-stem and TψC-stem), suggesting drastic changes that may affect the structure and function of mt-tRNAs.

**Figure 2:**
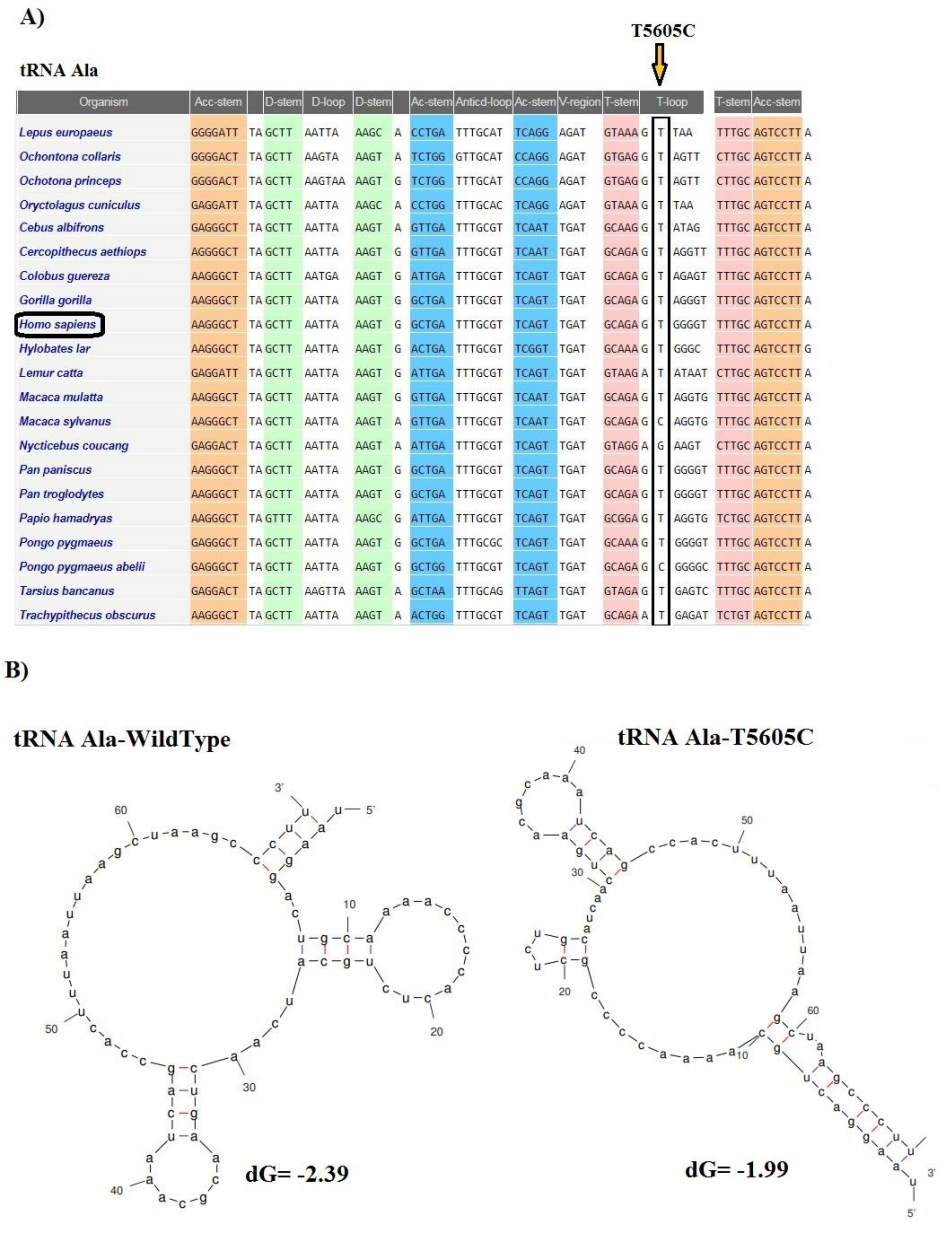
Sequence alignment of tRNA^Ala^ reveals a high-degree of conservation across species. (A) The T at the position 5605 nucleotide is highly conserved among species. (B) Prediction of the structural and folding changes due to a T5605C mutation in tRNA^Ala^ using the Mfold program. The free energy change between the mutant and wild-type states was determined.

**Figure 3:**
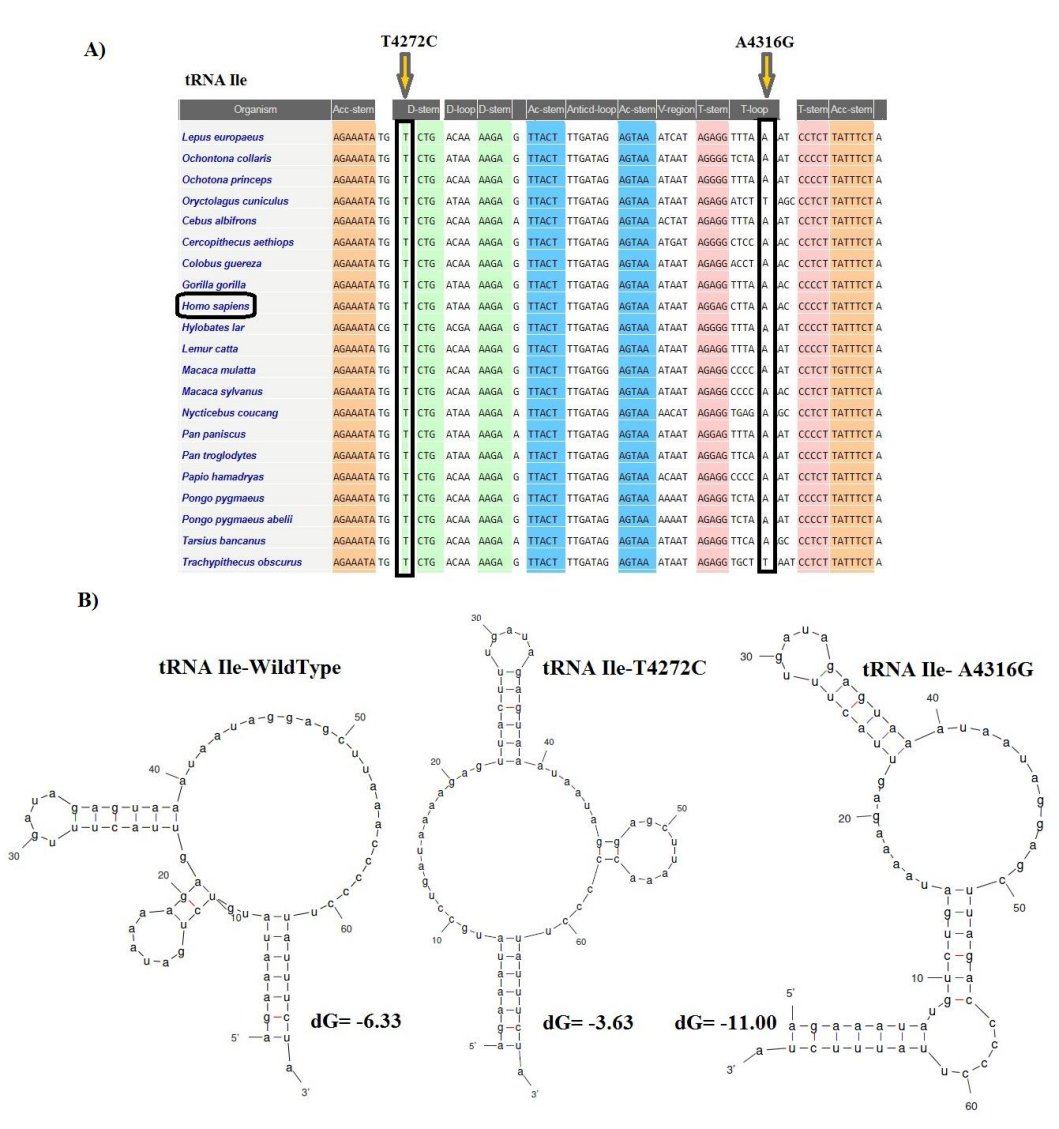
Sequence alignment of tRNA^Ile^ reveals a high-degree of conservation of T4272 and A4316 nucleotides across species. (A) The nucleotides T at the position 4272 and A at position 4316 are highly conserved among species. (B) Prediction of the structural and folding changes due to T4272C and A4316G nucleotides in tRNA^Ile^ using the Mfold program. The free energy change between the mutant and wild-type states was determined.

**Figure 4:**
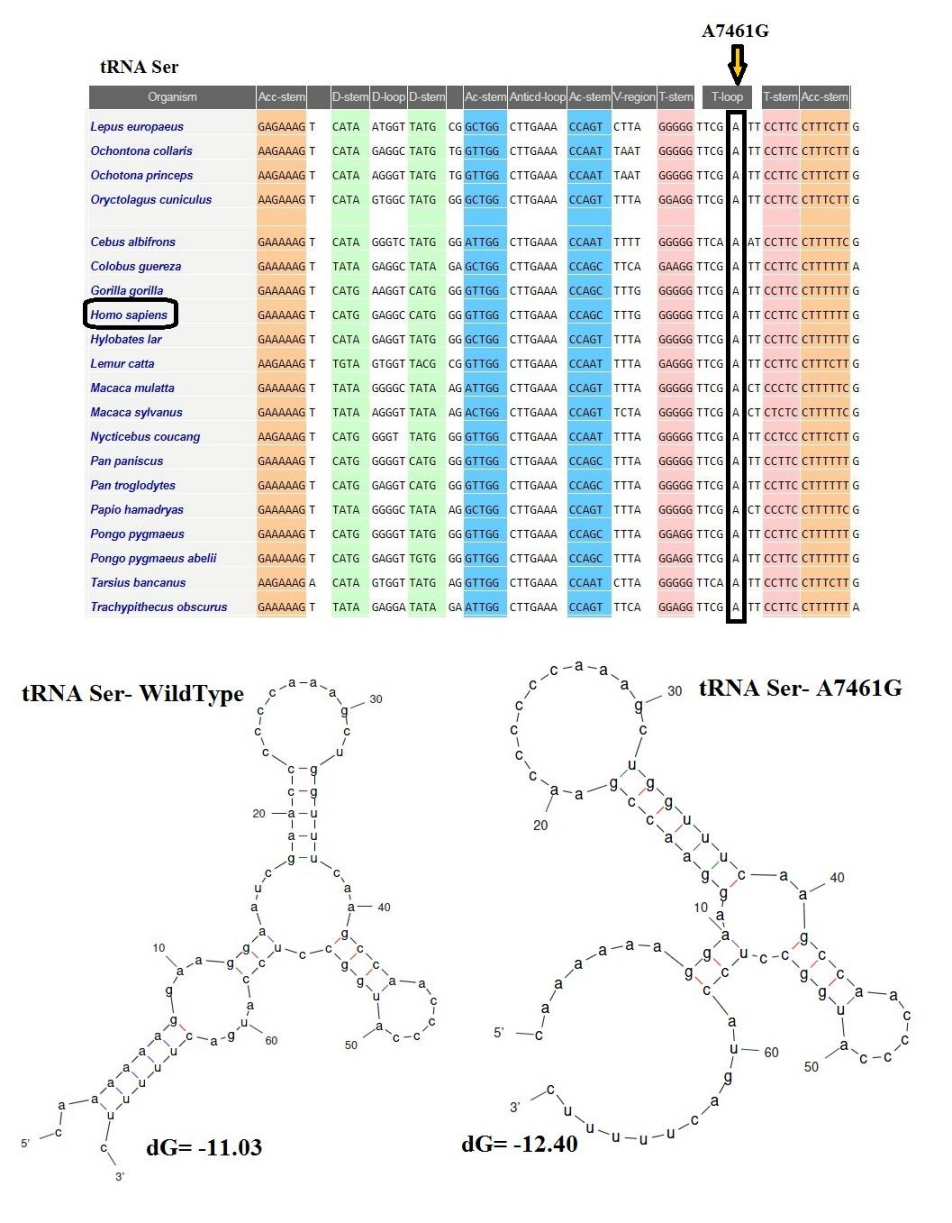
Sequence alignment of tRNA^Ser^ reveals a high-degree of conservation of A7461 nucleotide across species. A representation of the structural and folding effect of the A7461G mutation in tRNA^Ser^ was predicted using the Mfold program is also represented below the sequence alignment. The free energy change between the mutant and wild-type states was determined.

**Figure 5:**
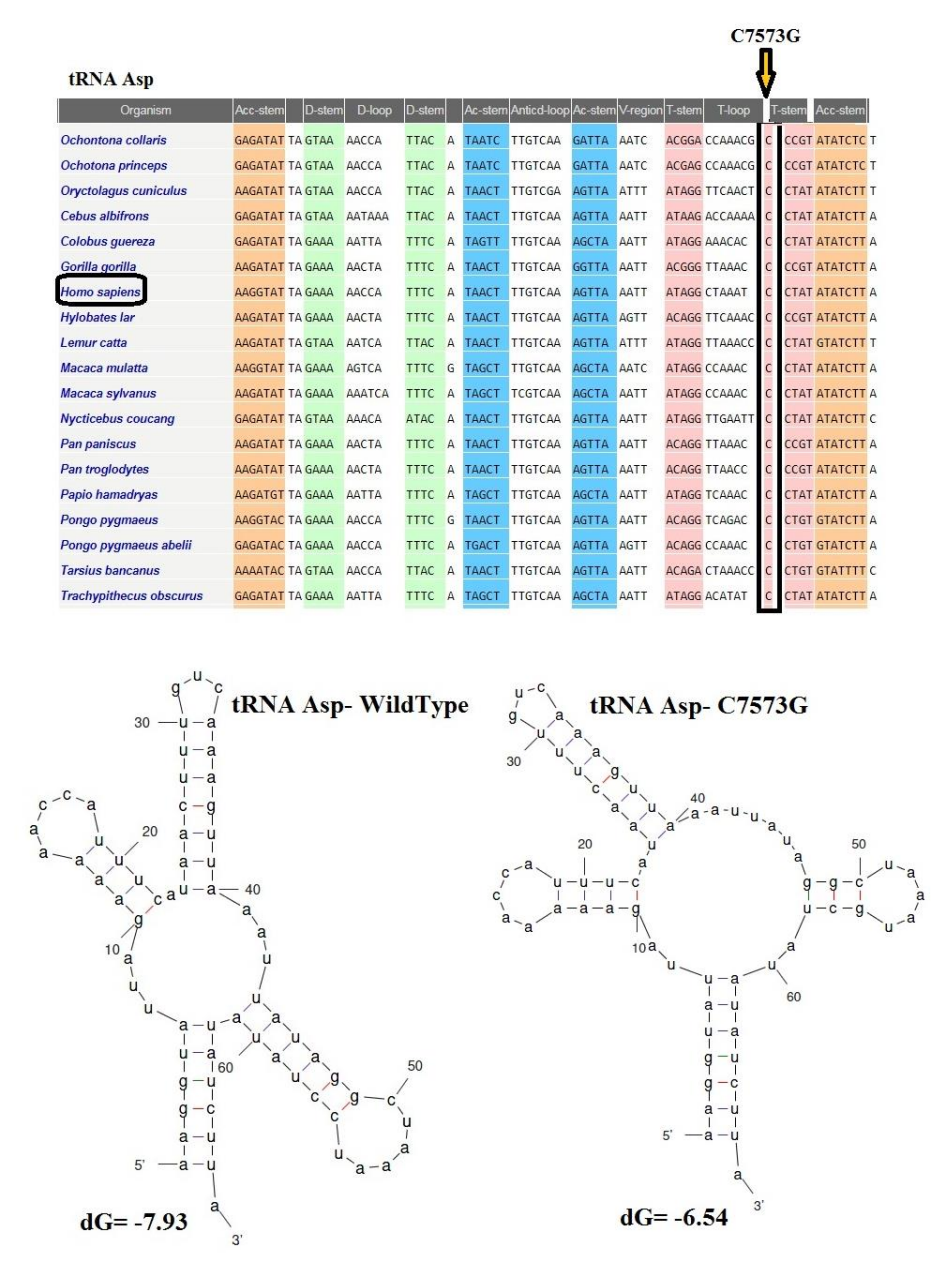
Sequence alignment of tRNA^Asp^ reveals a high-degree of conservation of C7573 nucleotide across species. A representation of the structural and folding effect of the C7573G mutation in tRNA^Asp^ was predicted using the Mfold program is also represented below the sequence alignment. The free energy change between the mutant and wild-type states was determined.

The CI analysis, MitoTIP, and the mfold structural predictor suggested that 8 mt-tRNA nucleotide variants, which had high conservation index and were not detected in healthy controls, may be pathogenic/likely pathogenic mutations. The conservation index of these mt-tRNA variants were as follows: T4272C in *tRNA^Ile^*: 99.00 %, A4316G in *tRNA^Ile^*: 90.47 %, T5543C in *tRNA^Trp^*: 99.00 %, T5605C in *tRNA^Ala^*: 85.71 %, A5656G in *tRNA^Ala^*: 99.00 %, T5670C in *tRNA^Asn^*: 76.19 %, G5727T in *tRNA^Asn^*: 99.00 %, A7461G in *tRNA^Ser^*: 99.00 %, C7573G in *tRNA^Asp^*: 99.00 %, and C8346T in *tRNA^Lys^*: 54.17 %. We observed that except for the C8346T in *tRNA^Lys^*, other variants in tRNA genes showed higher levels of CIs (CI≥75 %). Interestingly, other mt-tRNA variants were not detected in healthy controls and predicted to change the structure or function of tRNA molecules, suggesting that they may be involved in the pathogenesis of CHD. Considering that the evolutionary conservation of the variations is only observed in less than 1 % of the control group, we identify 9 pathogens/possibly pathogens in mt-tRNA in 23 patients with CHD, which can cause functional or structural changes in mt-tRNA molecules. Among them, only five mtDNA-tRNA mutations were found to change the free energy for the thermodynamic steady-state of the secondary structures of tRNAs; T4272C and A4316G in *tRNA^Ile^*, T5605C in *tRNA^Ala^*, A7461G in *tRNA^Ser^*, and C7573G in *tRNA^Asp ^*(Figures 2-5(Fig. 2)(Fig. 3)(Fig. 4)(Fig. 5)).

### Mitochondrial ATP measurement

**Figure 6:**
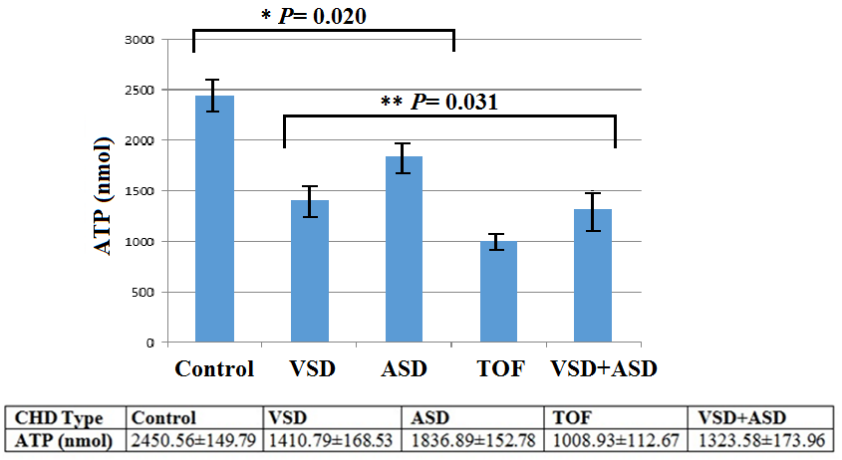
Bar diagram showing the intracellular ATP content in lymphocytes from healthy control and CHD patients (VSD, ASDS, TOF, and VSD+ASD). Intracellular ATP (nmol) was significantly reduced in patients with various type of CHD compared to the control group (*: significance line between patients and control group: *P*= 0.020 and **: significance line between CHD patients: *P*= 0.031).

After identifying point nucleotide variations in the mtDNA of CHD patients, we followed up on their biological significance in cells bearing these mutations by measuring the intracellular concentration of adenosine triphosphate (ATP) by preparing an ATP calibration curve. The cellular ATP content was significantly lower in the patients' groups (VSD, ASD, TOF, VSD+ASD, and ASD+TOF) than in healthy controls (*P*= 0.020). Mean value for patients (n=200) and control subjects (n=160) were 1395.04 ± 151.98 and 2450.56 ± 149.79 (Mean±SD), respectively (Figure 6(Fig. 6)). 

## Discussion

Most research groups have addressed the risk factors associated with mutations occurring in the nuclear genome and CHD, while only a few studies were undertaken to understand the role of mitochondrial DNA mutations. Interestingly, pathological mutations in the mitochondrial genome were reported to be major causes of cardiovascular disease (Poznyak and Ivanova, 2020[34]). Determining the effect of mitochondrial DNA mutations on energy production and mitochondrial dysfunction would improve the understanding of heart development. The nucleotide changes in the mtDNA were reported to lead to important defects in the concentration of several respiratory enzymes and transfer RNAs (tRNAs) directly synthesized in the mitochondria (Mayr et al., 2015[31]; Liu and Chen, 2020[29]). Moreover, some inherited mtDNA polymorphisms that are not directly related to any form of pathology have been shown to affect mitochondrial function (Bray and Ballinger, 2017[8]). Cardiovascular disorders are often caused by multiple genetic mutations, and the severity of the clinical phenotype is often correlated to the number of mutations in the genome which may have an additive pathological effect (Kelly and Semsarian, 2009[25]). Production of energy by OXPHOS is performed by mitochondria, and some mutations in mtDNA genes were shown to affect the proper functioning of the heart cells (Siasos et al., 2018[39]). Most pathogenic mtDNA mutations are heteroplasmic and play important roles in mitochondrial dysfunction, leading to the development and severity exacerbation of many human disorders such as cardiac disorders (Kanungo et al., 2018[23]). Recent studies by Jia et al. (2013[20]), Qin et al. (2014[35]), Jiang et al. (2016[21]), Heidari et al. (2017[16]), and Schiattarella et al. (2014[38]) have identified point nucleotide variations in the mitochondrial *tRNA^Thr^, tRNA^Trp^, tRNA^Leu^, tRNA^Ile^, tRNA^Met^, tRNA^Phe^, tRNA^Ala^, *and* tRNA^Gln^* genes associated with both early onset coronary artery disease and hypertension. Here, we have analyzed mtDNA mutations in 200 pediatric patients with varying degrees of CHD severity and identified a total of 23 genetic variants in mt-tRNA and protein-coding genes. Ten nucleotide variations were located in the tRNA coding genes, consequently, these variations are not expected to cause any change in the amino acid sequences of proteins. However, we predicted that the mutations T4272C and A4316G in *tRNA^Ile^*, T5605C in *tRNA^Ala^*, A7461G in *tRNA^Ser^*, and C7573G in *tRNA^Asp ^*change the free energy in the secondary structure of tRNAs, which may affect either the specific accessibility to the appropriate mRNA codon that ensures the addition of correct amino acid during translation or the stability of the tRNAs, ultimately also causing a reduction of the activity of oxidative phosphorylation complexes. Most of the mt-tRNA variations that we report here are novel, but among those variants, two heteroplasmic changes were the most commonly associated with pathogenic mutations. Moreover, A4316G change in the TψC loop of tRNA^Ile^ occurs in a nucleotide extremely conserved across various species. Interestingly, A4316G change has been previously associated with hypertrophic cardiomyopathy syndrome (HCM) with hearing loss (Alves et al., 2016[2]), indicating that this mutation could be involved in the pathogenicity of more than one cardiovascular condition. The T5543C located in the anticodon-loop of tRNATrp was previously reported as a pathogenic variant in juvenile myopathy, encephalopathy, lactic acidosis, stroke, and mitochondrial myopathy (Anitori et al., 2005[4]). 

Interestingly, the novel and homoplasmic A5656G variant, that we identified here, is located at an extremely conserved position in the non-coding region just before the processing site of the 5ʹ end of tRNA^Ala^. Interestingly, precursor processing of mt-tRNAs requires a precise endonucleolytic cleavage at both 3ʹ and 5ʹ ends. At the 5ʹ end, extra nucleotides are removed with the 5´-tRNA cleavage by the mt-RNase P, while at the 3ʹ end; tRNA processing of the precursor transcript is catalyzed by 3'-endonuclease (Karasik et al., 2019[24]; Berg and Brandl, 2021[7]). Therefore, it is tempting to propose that the A-to-G transition occurring at position 5656 in the light strand of mtDNA may lead to a defective 5ʹ end processing in the primary transcript and correct cleavage of primary mt-tRNA transcripts into mature tRNA^Ala^. Interestingly, it has been shown that the 5ʹ and 3ʹ end processing deficiency, as a result of pathogenic mitochondrial tRNA mutations can be associated with many disorders (Tafti et al., 2018[42]). 

Moreover, the novel and heteroplasmic G5727T variant disrupted a G-C base pair within the acceptor stem of tRNA^Asn^, located at a highly conserved site (CI>95 %) and was observed in only three children with TOF. All three patients had severe symptoms and underwent surgery. Furthermore, the A7461G variant was identified in the TψC loop of the tRNA^Ser^ (UCN) gene, this variant occurred at a highly conserved base-pairing (A54-U65) of tRNA^Ser^ (UCN), which is significant for related aminoacyl tRNA synthetase recognition. The destruction of A54-U65 Watson-Crick base-pairing probably impaired the tRNA^Ser^ (UCN) metabolism and affected the mt-tRNA function. Additionally, the C7573G variant was observed in the tRNA^Asp^ gene, which disrupted the highly conserved (CI>95 %) Watson-Crick base-pairing (C56-G49). Therefore, the alteration of mt-tRNA structure and change in the free energy for the thermodynamic steady-state of the tRNAAsp caused by this variant subsequently can lead to failure in mt-tRNA metabolism and its function.

We also identified thirteen nucleotide variations in the protein-coding genes (*COXI, COXII, ATPase8, *and* ATPase6*). Some nucleotide changes in the mitochondrial genome are non-synonymous mutations, causing amino acid changes, and may have a remarkable effect on protein structure and function. Although mitochondrial synonymous polymorphisms are thought to have no pathogenic effect, these variants may have negative effects on the efficiency of gene expression (Hunt et al., 2014[19]). We presented here five mitochondrial missense mutations, and at least three of them were considered pathogenic variations. The most remarkable mtDNA candidate genes in cardiac diseases, besides tRNA genes, are COXI, COXII, COXIII (complex IV), ATPase 6, and ATPase 8 (complex V). Several studies showed that missense mutations in these mitochondrial genes are associated with various cardiac and muscular diseases (Jonckheere et al., 2009[22]; Chistiakov et al., 2012[9]). Sobenin et al. suggested that some mtDNA missense mutations may be potential causes of atherosclerosis development in humans. They showed that these mutations had a higher prevalence in atherosclerotic samples, and thus can be used as genetic markers in coronary heart disease (Sobenin et al., 2012[40]). Heidari et al. (2020[18]), and Zhu et al. (2009[50]) independently showed that novel point mutations 8231C>A in *mt-COXII*, 8376T>A and 8414C>T in *mt-ATPase8*, and 8701A>G and 8584G>A in *mt-ATPase6* genes are linked with coronary artery disease and hypertension. Several studies proposed that the mtDNA mutations lead to dysfunctions of OXPHOS in the mitochondria, increase ROS generation, reduction of the efficiency of energy production and mitochondrial protein synthesis, interrupt normal sodium and calcium metabolism, dysfunctional the diastolic function in cardiomyocytes, and finally cardiac disorders (Ramaccini et al., 2021[36]; Dabravolski and Khotina, 2022[10]). 

In the present study, five missense mutations were detected in *COXI* and *ATPase6* genes encoding proteins that play important roles in electron transfer in mitochondria during ATP synthesis. Here, ConSurf analysis revealed that the C7398T (Pro499Ser) and C7418G (Phe505Leu) variants in the *COXI* gene presented very high evolutionary conservation, and were absent in the 160 Iranian healthy controls. Interestingly, these non-synonymous variations have not been reported previously on the MITOMAP site and were identified for the first time in CHD. The Pro499Ser variation alters a highly conserved Proline codon (CCC), a hydrophilic amino acid (hydrophobicity score: -1.600), to Serine codon (UCC), a polar amino acid (hydrophobicity score: -0.800) that was found in 2 patients with VSD. Also, Phe505Leu variation changes a highly conserved Phenylalanine codon (UUC), an aromatic and very hydrophobic amino acid (hydrophobicity score: 2.800), to Leucine codon (UUG), an aliphatic and very hydrophobic amino acid (hydrophobicity score: 3.800) that was found in 5 patients with ASD and VSD. These substitutions are predicted to be probably damaging and to affect protein function by the PolyPhen-2 prediction method with scores of (0.999) and 0.995, respectively. 

Cytochrome C oxidase subunit 1 (COXI) is a transmembrane protein and one of the three mtDNA encoded subunits of the respiratory Complex IV. Three critical enzymes of this complex including COXI, COXII, and COXIII form the catalytic and functional core of the enzyme complex and their structure and functions have been highly conserved in different species of eukaryotes. Therefore, amino acid substitutions in the *mt-COXII* gene are identified as important causes of cytochrome C oxidase deficiency and consequently mitochondrial complex IV deficiency which was associated with various medical disorders in different tissues (Timón-Gómez et al., 2018[43]). 

Mitochondrial complex V or F0F1-ATP synthase comprises two practical domains, F1 and F0, and is positioned in the mitochondrial inner membrane and is critical for synthesizing ATP in mitochondria. The F0 fragment contains three to nine subunits of which only subunits 6 and 8 are encoded by mitochondrial *mt-ATPase6* and *mt-ATPase8* genes, respectively. Considering the extraordinary importance of complex V, it is not surprising that amino acid changes in this enzyme complex have destructive effects in energy-dependent tissues and changes in the metabolic stability of the cell energy state and so lead to mitochondrial disorders (Mnatsakanyan and Jonas, 2020[33]). 

Here, we reported a homoplasmic m.8860A>G (Thr112Ala) mutation and two heteroplasmic m.8878C>G (Arg118Gly) and m.9041A>G (His172Arg) mutations in *mt-ATP6* gene that was found for the first time in CHD. The novel m.8860A>G mutation converted the neutral Threonine (hydrophobicity score: -0.700) at a conserved amino acid position 112 to hydrophobic Alanine (hydrophobicity score: 1.800) in the transmembrane region of ATPase 6 protein (Figure 2(Fig. 2)) and was detected in 3 CHD patients (2.5 %) and absent in our healthy controls (*P*=0.011). This mutation has not been reported previously in any disease. In addition, the m.9041A>G mutation converted the neutral Histidine (hydrophobicity score: -3.200) at a conserved amino acid position 172 to hydrophilic Arginine (hydrophobicity score: -4.500) in the matrix domain of ATPase 6 protein and was detected in 4 CHD patients (3.5 %) and was absent in our healthy controls (*P*=0.001). This mutation was found for the first time in CHD patients but was previously reported as a pathogenic variant by Ganetzky et al. in muscle weakness, exercise intolerance, and congenital cataracts (Ganetzky et al., 2019[12]). 

**Figure 7:**
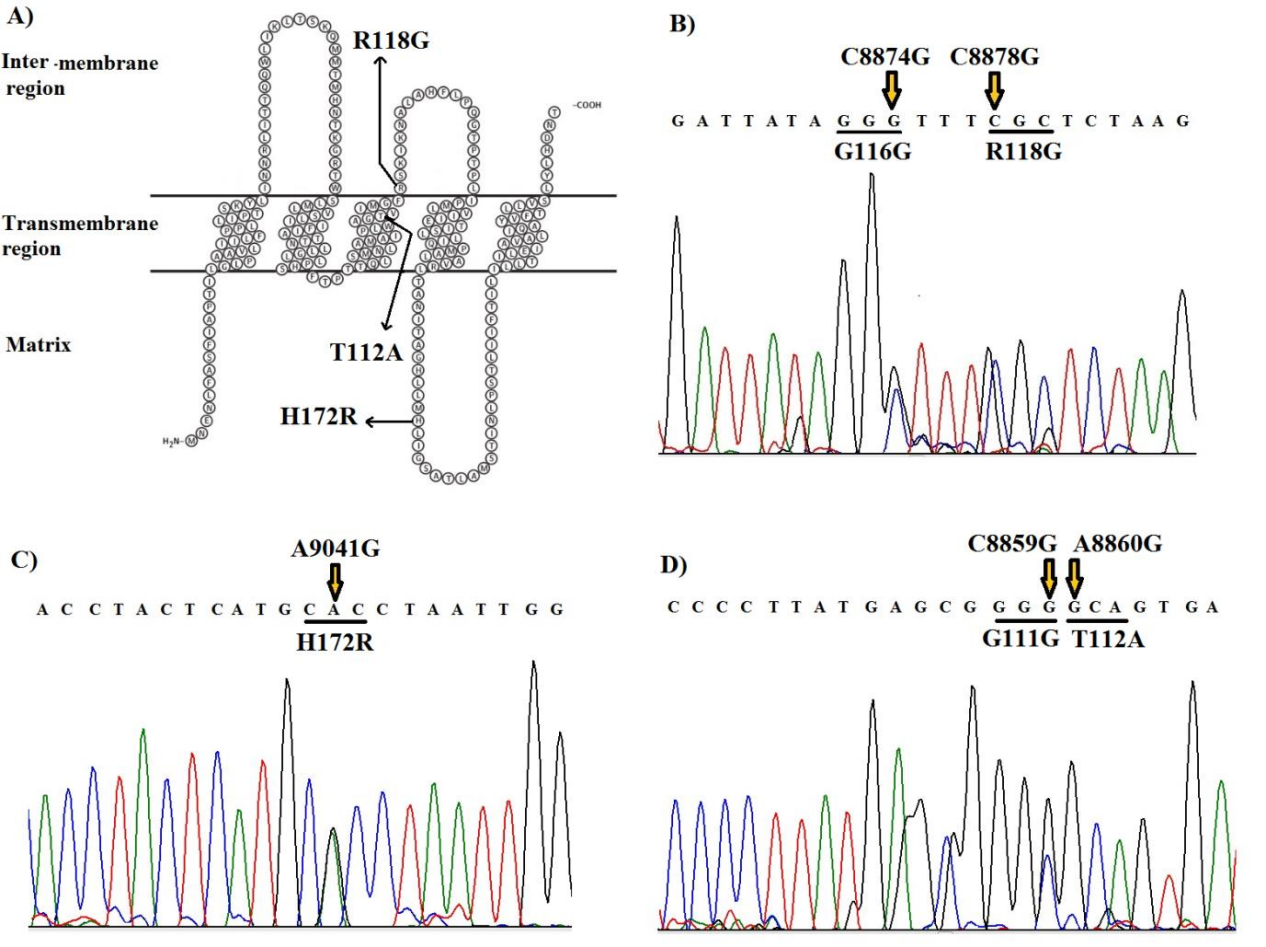
A) The position of three missense mutations (Thr112Ala, Arg118Gly, and His172Arg) and changes of related amino acids in the secondary structure of the ATPase6 protein, B, C, and D) Sequencing results of three heterozygous missense variations in *ATPase6* gene in CHD patients. Two patients with VSD symptoms showed two nucleotide variations in the ATPase6 gene, simultaneously: C8859G (Gly111Gly) and A8860G (Thr112Ala) variations in a 1.5-years-old girl, and C8874G (Gly116Gly) and C8878G (Arg118Gly) variations in a 6-months-old boy.

We also detected two patients, a 1.5-years-old girl, and a 6-months-old boy, who are clinically diagnosed with ventricular septal defect (VSD) with symptoms such as difficulty feeding, rapid breathing, sweating while eating, and slow weight gain. The results of the mitochondrial genome study of these two patients showed that both carry two nucleotide variations in the ATPase6 gene, simultaneously: heteroplasmic C8859G (Gly111Gly) and homoplasmic A8860G (Thr112Ala) variations in a 1.5-years-old girl, and heteroplasmic C8874G (Gly116Gly) and C8878G (Arg118Gly) variations in a 6-months-old boy (Figure 7(Fig. 7)). All these four variants were novel, and Gly111Gly and Gly116Gly variations were seen in 5 and 6 of 200 cases (2.5 % and 3 %), respectively, but were not present in the control subjects (*P*=0.006). The heteroplasmic missense mutations in the ATPase6 gene (Thr112Ala and Arg118Gly) were not yet reported as neutral variations. Moreover, these variants were not observed in the control individuals with the same ethnic backgrounds in our study. Given that the heart defects of these two patients were relatively similar to each other, clinical findings suggest that heart failure in both patients may result from a synergic effect of these variations on the molecular pathogenesis subjacent to CHD.

We then established that the generation of ATP was reduced in cells harboring mtDNA point mutation variants in comparison to the control, suggesting that variations impair the function of the respiration chain and inhibit mitochondrial ATP synthesis. Recent studies have shown that the mt-tRNA point mutations and missense mutations resulted in a significant reduction of the activity of mitochondrial complexes I, III, IV, and V, leading to lower levels of ATP production (Ganetzky et al., 2019[12]; Liu and Chen, 2020[29]). Cells from patients with TOF who carried the 4316AG (in T-loop tRNA^ILe^), 5543T>C (in the anticodon loop of tRNA^Trp^), 5727G>T (in ACC-stem of tRNA^Asn^), 8878C>G, 8874C>G (Arg118Gly, Gly116Gly in ATPase6) mutations had 58.83 % lower ATP generation level than control cells. Cells from patients with ASD+VSD who carried the 4272T>C (in D-Stem of tRNA^Ile^), 5605T>C (in T-loop tRNA^Ala^), 5656A>G (processing site in 5ʹ-end of tRNA^Ala^), mutations had 45.98 % lower ATP generation levels. Similarly, in cells from patients with VSD and ASD, ATP levels were 42.42 % and 25.04 % lower than in normal cells, respectively. Thus, mtDNA point mutations that have structural and functional effects on tRNA or coding genes probably cause a decrease in the tRNA or mRNA steady-state level, contributing to mitochondrial dysfunction as well as impaired mitochondrial translation, and the decline of ATP production which may in turn lead to the progression and pathogenesis of cardiovascular cell defects, in particular, CHD.

**Figure 8:**
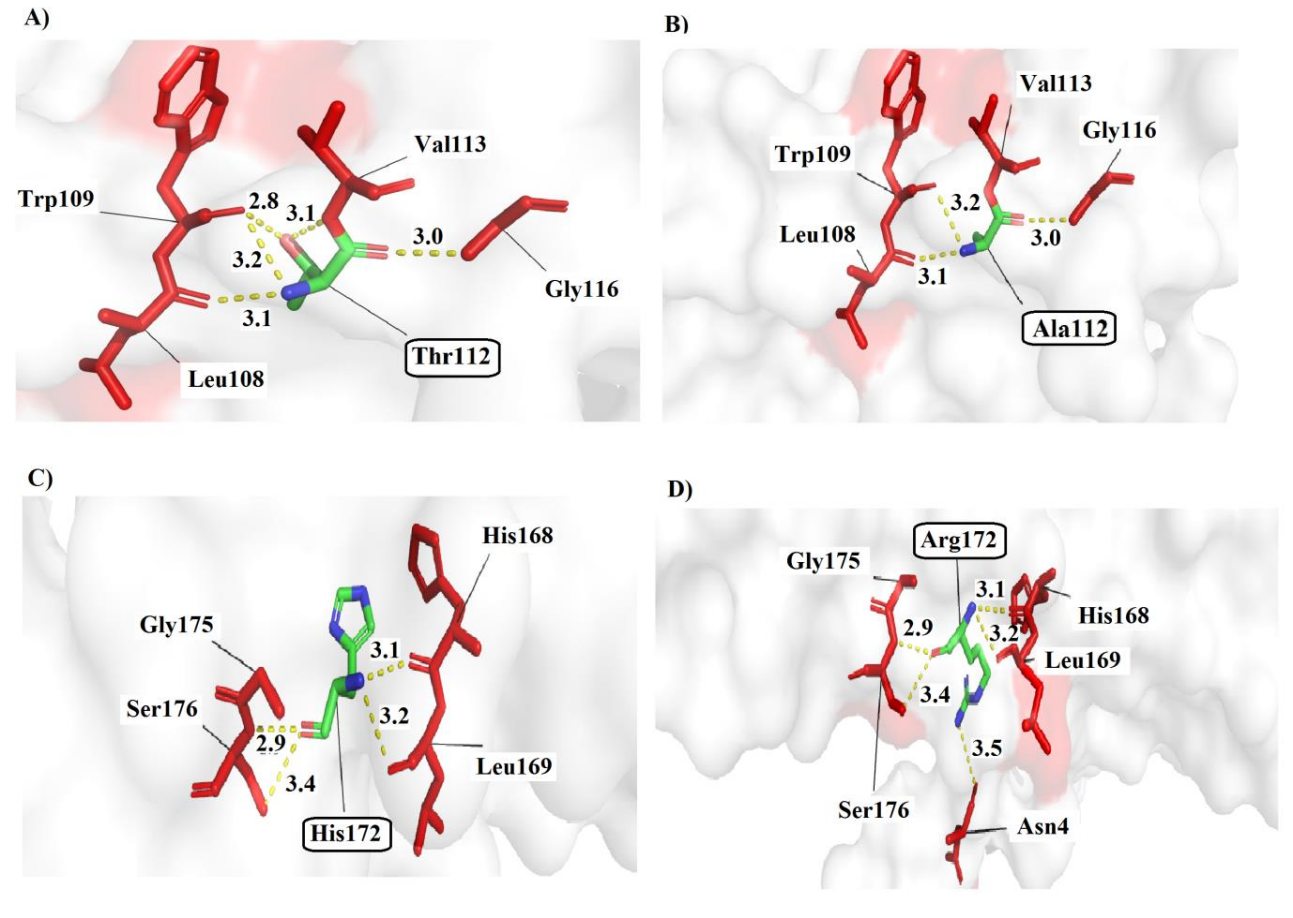
Three-dimensional structure model predictions for normal and mutant variants; A, B) Thr112 (Normal) and Ala112 (mutant), C, D) His172 (Normal) and Arg172 (mutant) in ATPase6 by PyMol software

Our structural human ATPase6 protein modeling resulting from SWISS-MODEL, PyMol, and 3-D docking analysis further showed that m.8860A>G (Thr112Ala) mutation is predicted to affect protein structure. Indeed, the Threonine in the third alpha helix of the transmembrane region in ATPase6 interacts with amino acids Val113 (3.1 A^ₒ^), Trp109 (2.8 and 3.2 A^ₒ^), and Leu108 (3.1 A^ₒ^), with four polar contacts, while in the mutated protein version, the Alanine interacts with Trp109 (3.2 A^ₒ^), Leu108 (3.1 A^ₒ^), and Gly116 (3.0 A^ₒ^) with three polar contacts (Figure 8(Fig. 8)). Although the SIFT and Polyphen analysis predicted the p.Thr112Ala variant was found to be tolerated and benign with scores of 0.21 and 0.000, respectively, PROVEAN and PANTER prediction results showed that this variant was deleterious and probably damaging (score=0.85). However, Thr112 is a structural significant residue involved in the transmembrane functional domain of ATPase6 protein and therefore the substitution of Threonine to Alanine affects the structure of this functional domain and possibly changes the function of the protein.

**Figure 9:**
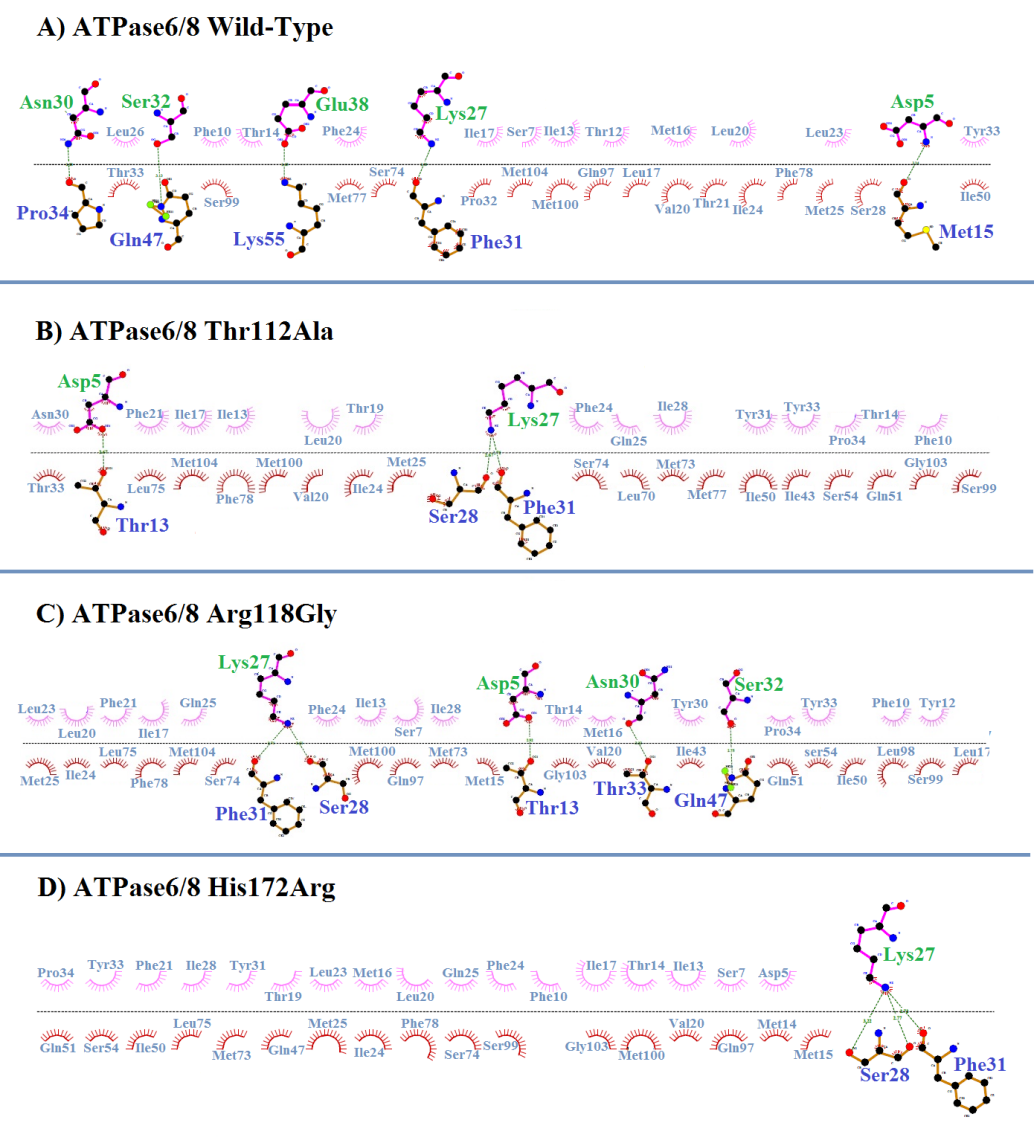
A-D) Comparison of hydrogen bonding patterns and hydrophobic interaction patterns between wild-type and mutant states in ATPase6 by structural and molecular docking between ATPase6 and ATPase8 amino acids. Hydrogen bonds are shown by dashed lines (green) between wild-type and mutant states of ATPase6 (upper line) with ATPase8 (bottom line) residues and hydrophobic interactions are indicated by feathered arcs between residues.

Another transition G-to-A in position 9041 of the *mt-ATP6* gene, converting the Histidine at position 172 to an Arginine in the functional matrix domain of ATPase6 protein (p.His172Arg) (Figure 8(Fig. 8)), Polyphen, PROVEAN, PANTER, and PredictSNP analysis predicted the p.His172Arg variant to be “Possibly damaging” (score=0.464), “Deleterious”, and “Probably damaging” (score= 0.50). Also, in p.His172Arg missense mutation, Histidine in the normal state formed four polar interactions with amino acids His168 (3.1 A^ₒ^), Leu169 (3.2 A^ₒ^), Gly175 (2.9 A^ₒ^), and Ser176 (3.4 A^ₒ^) while in the mutant state, Arginine formed five interactions with His168 (3.1 A^ₒ^), Leu169 (3.2 A^ₒ^), Gly175 (2.9 A^ₒ^), and Ser176 (3.4 A^ₒ^), and Asn4 (3.5 A^ₒ^). Structural modeling analysis for three other missense mutations in *COXI* and *ATPase6* genes in our patients did not show obvious changes in protein geometry (data not shown). Finally, DIMPLOT program plots for protein-protein or domain-domain interactions in normal ATPase6 and ATPase8 proteins showed the presence of several residue-residue polar and hydrophobic interactions that will change with three missense mutations in ATPase6 (Thr112Ala, Arg118Gly, and His172Arg) (Figure 9(Fig. 9)). DIMPLOT analysis revealed ten amino acids' involvement in forming five hydrogen bonds in the wild-type interactions between ATPase6 and ATPase8. In contrast, in the mutant forms of the ATPase6 protein, the number, and type of amino acids involved in the hydrogen interactions with ATPase8 protein were drastically changed.

In recent studies, the missense mutations in the human *mt-COXI*, *mt-COXII*, *mt-ATPase6,* *mt-ATPase8, *and genes have often been presented as pathogenic variants, since they are associated with defective mitochondrial complex IV and V, and their pathogenicity is estimated by important criteria such as the evolutionary conservation of the altered amino acid as well as the percentage prevalence of these mutations in control populations (Andreu et al., 2000[3]; Arena et al., 2022[5]).

## Conclusion

To our knowledge, this is the first study that evaluates the relationship between nucleotide variations in several mt-coding and mt-tRNA genes (about 33.15 % of mtDNA) with CHD risk in an Iranian population. Here, we identify 23 nucleotide changes, including 17 novel mitochondrial mutations that probably affect the structure and function of the studied mitochondrial complexes. These nucleotide changes suggest the role of mitochondrial mutations as predisposing factors that influence the pathogenesis of CHD. However, more research is needed to better understand the pathogenesis and the predisposing effects of these changes on cardiovascular disease. The study presented here provides an open window to expand current methods and reveal new interactions that could underlie disease-associated relationships between congenital heart defects and mitochondrial genome disorders.

## Declaration

### Acknowledgments 

We would like to thank all the patients who cooperated with us in preparing blood samples for scientific investigations, as well as the medical staff of Afshar Special Hospital (Yazd, Iran).

### Author contributions

M.M.H. and M.Kh. conceived and designed the study and also drafted the initial manuscript. A.K., M.Ka., M.M. and M.H.E. collected and analyzed the data. M.Kh., M.M.H. and J.B. performed statistics and interpretation of data and drafted the final version of the manuscript. M.N. and B.M. contributed to the writing of the manuscript and performed part of the bioinformatic analysis. All authors read and approved the final manuscript.

### Disclosure statement

The authors report no conflicts of interest.

### Financial disclosure 

There is no financial disclosure. 

## Figures and Tables

**Table 1:**
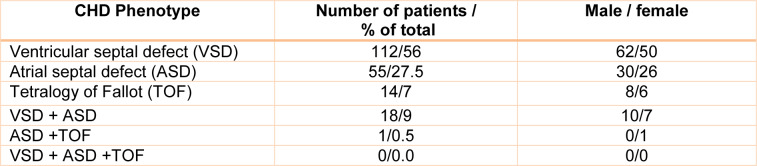
Clinical characteristics of unrelated non-syndromic CHD patients

**Table 2:**
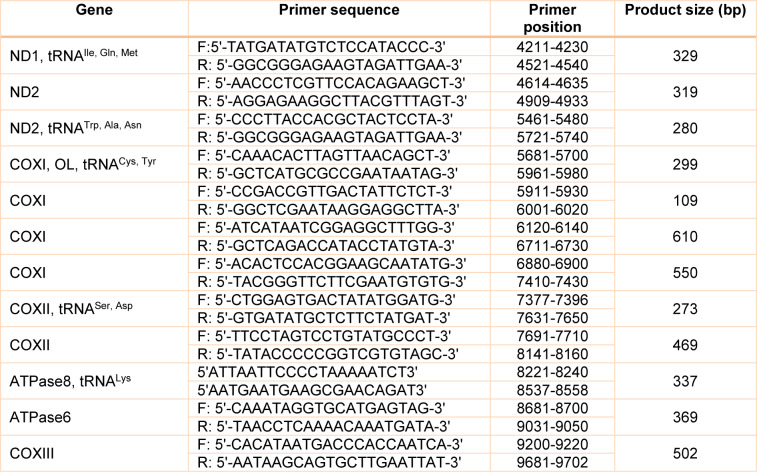
Specific primer sequences used for analysis of selected mitochondrial genes

**Table 3:**
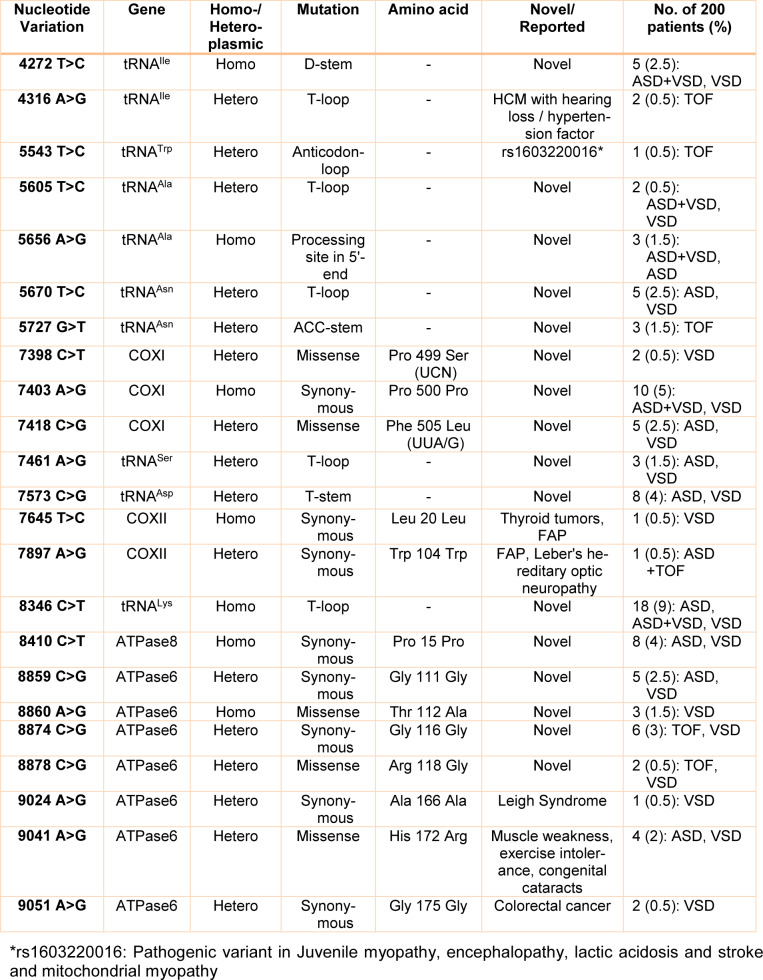
Nucleotide variations in mtDNA observed in the selected coding regions and tRNA genes

**Table 4:**
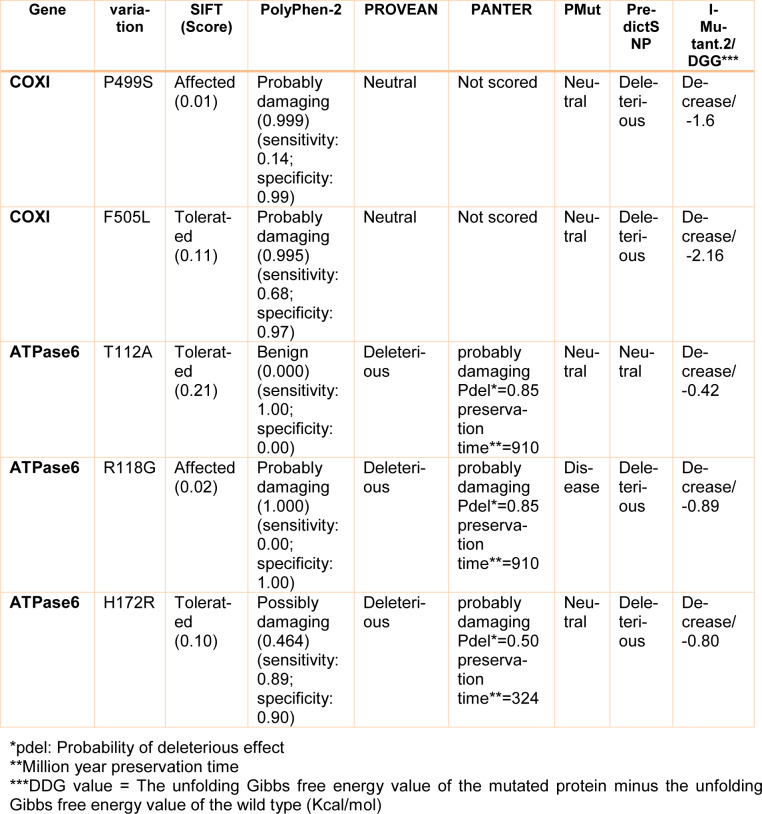
Prediction results for 5 missense mutations in *COXI* and *ATPase6* genes by functional and structural prediction tools

## References

[1] Abaci N, Arıkan M, Tansel T, Sahin N, Cakiris A, Pacal F (2015). Mitochondrial mutations in patients with congenital heart defects by next generation sequencing technology. Cardiol Young.

[2] Alves RM, da Silva Costa SM, do Amôr Divino Miranda PM, Ramos PZ, Marconi TG, Santos Oliveira G (2016). Analysis of mitochondrial alterations in Brazilian patients with sensorineural hearing loss using MALDI-TOF mass spectrometry. BMC Med Genet.

[3] Andreu AL, Checcarelli N, Iwata S, Shanske S, DiMauro S (2000). A missense mutation in the mitochondrial cytochrome b gene in a revisited case with histiocytoid cardiomyopathy. Pediatr Res.

[4] Anitori R, Manning K, Quan F, Weleber R, Buist N, Shoubridge E (2005). Contrasting phenotypes in three patients with novel mutations in mitochondrial tRNA genes. Mol Genet Metabol.

[5] Arena IG, Pugliese A, Volta S, Toscano A, Musumeci O (2022). Molecular genetics overview of primary mitochondrial myopathies. J Clin Med.

[6] Bates MG, Bourke JP, Giordano C, d'Amati G, Turnbull DM, Taylor RW (2012). Cardiac involvement in mitochondrial DNA disease: clinical spectrum, diagnosis, and management. Eur Heart J.

[7] Berg MD, Brandl CJ (2021). Transfer RNAs: diversity in form and function. RNA Biol.

[8] Bray AW, Ballinger SW (2017). Mitochondrial DNA mutations and cardiovascular disease. Curr Opin Cardiol.

[9] Chistiakov DA, Sobenin IA, Bobryshev YV, Orekhov AN (2012). Mitochondrial dysfunction and mitochondrial DNA mutations in atherosclerotic complications in diabetes. World J Cardiol.

[10] Dabravolski SA, Khotina VA (2022). The role of mitochondrial DNA mutations in cardiovascular diseases. Int J Mol Sci.

[11] Doenst T, Nguyen TD, Abel ED (2013). Cardiac metabolism in heart failure: implications beyond ATP production. Circ Res.

[12] Ganetzky RD, Stendel C, McCormick EM (2019). MT-ATP6 mitochondrial disease variants: Phenotypic and biochemical features analysis in 218 published cases and cohort of 14 new cases. Hum Mutat.

[13] Gomes KP, Jadli AS, de Almeida LGN, Ballasy NN, Edalat P, Shandilya R (2022). Proteomic analysis suggests altered mitochondrial metabolic profile associated with diabetic cardiomyopathy. Front Cardiovasc Med.

[14] Habbane M, Montoya J (2021). Human mitochondrial DNA: Particularities and diseases. Biomedicines.

[15] Hayes-Lattin M, Salmi D (2020). Educational case: tetralogy of fallot and a review of the most common forms of congenital heart disease. Acad Pathol.

[16] Heidari MM, Derakhshani M, Sedighi F, Foruzan-Nia SK (2017). Mutation analysis of the mitochondrial tRNA genes in Iranian coronary atherosclerosis patients. Iran J Public Health.

[17] Heidari MM, Houshmand M, Nafissi S, Khatami M (2009). Complex I and ATP content deficiency in lymphocytes from Friedreich's ataxia. Can J Neurol Sci.

[18] Heidari MM, Mirfakhradini F, Tayefi F, Ghorbani S, Khatami M, Hadadzadeh M (2020). Novel point mutations in mitochondrial MT-CO2 gene may be risk factors for coronary artery disease. Appl Biochem Biotechnol.

[19] Hunt R, Simhadri V, Iandoli M, Sauna Z, Kimchi-Sarfaty C (2014). Exposing synonymous mutations. Trends Genet.

[20] Jia Z, Wang X, Qin Y, Xue L, Jiang P, Meng Y (2013). Coronary heart disease is associated with a mutation in mitochondrial tRNA. Hum Mol Genet.

[21] Jiang P, Wang M, Xue L, Xiao Y, Yu J, Wang H (2016). A Hypertension-associated tRNAAla mutation alters tRNA metabolism and mitochondrial function. Mol Cell Biol.

[22] Jonckheere AI, Hogeveen M, Nijtmans LG, van den Brand MA, Janssen AJ, Diepstra JH (2008). A novel mitochondrial ATP8 gene mutation in a patient with apical hypertrophic cardiomyopathy and neuropathy. J Med Genet.

[23] Kanungo S, Morton J, Neelakantan M, Ching K, Saeedian J, Goldstein A (2018). Mitochondrial disorders. Ann Transl Med.

[24] Karasik A, Fierke CA, Koutmos M (2019). Interplay between substrate recognition, 5' end tRNA processing and methylation activity of human mitochondrial RNase P. RNA.

[25] Kelly M, Semsarian C (2009). Multiple mutations in genetic cardiovascular disease. Circ Cardiovasc Genet.

[26] Khatami M, Heidari MM, Karimian N, Hadadzadeh M (2016). Mitochondrial mutations in tRNAGlu and cytochrome b genes associated with Iranian congenial heart disease. Int Cardiovasc Res J.

[27] Kloesel B, DiNardo JA, Body SC (2016). Cardiac embryology and molecular mechanisms of congenital heart disease: a primer for anesthesiologists. Anesth Analg.

[28] Laskowski RA, Swindells MB (2011). LigPlot+: Multiple ligand–protein interaction diagrams for drug discovery. J Chem Inf Model.

[29] Liu Y, Chen Y (2020). Mitochondrial tRNA mutations associated with essential hypertension: from molecular genetics to function. Front Cell Dev Biol.

[30] Lopaschuk GD, Karwi QG, Tian R, Wende AR, Abel ED (2021). Cardiac energy metabolism in heart failure. Circ Res.

[31] Mayr JA, Haack TB, Freisinger P, Karall D, Makowski C, Koch J (2015). Spectrum of combined respiratory chain defects. J Inherit Metab Dis.

[32] Mazzaccara C, Mirra B, Barretta F, Caiazza M (2021). Molecular epidemiology of mitochondrial cardiomyopathy: a search among mitochondrial and nuclear genes. Int J Mol Sci.

[33] Mnatsakanyan N, Jonas EA (2020). The new role of F1Fo ATP synthase in mitochondria-mediated neurodegeneration and neuroprotection. Exp Neurol.

[34] Poznyak AV, Ivanova EA (2020). The role of mitochondria in cardiovascular diseases. Biology (Basel).

[35] Qin Y, Xue L, Jiang P, Xu M, He Y, Shi S (2014). Mitochondrial tRNA variants in Chinese Subjects with coronary heart disease. J Am Heart Assoc.

[36] Ramaccini D, Montoya-Uribe V, Aan FJ, Modesti L, Potes Y, Wieckowski MR (2021). Mitochondrial function and dysfunction in dilated cardiomyopathy. Front Cell Dev Biol.

[37] Saker PJ (1998). Mutation screening using PCR-SSCP: silver staining and isotopic protocols. Methods Mol Med.

[38] Schiattarella GG, Trimarco B, Perrino C, Esposito G (2014). tURn the lights on: mitochondrial transport-RNAs and cardiovascular disease. J Am Heart Assoc.

[39] Siasos G, Tsigkou V, Kosmopoulos M, Theodosiadis D, Simantiris S, Tagkou NM (2018). Mitochondria and cardiovascular diseases-from pathophysiology to treatment. Ann Transl Med.

[40] Sobenin IA, Sazonova MA, Ivanova MM, Zhelankin AV, Myasoedova VA, Postnov AY (2012). Mutation C3256T of mitochondrial genome in white blood cells: novel genetic marker of atherosclerosis and coronary heart disease. PLoS One.

[41] Sonney S, Leipzig J, Lott MT, Zhang S, Procaccio V, Wallace DC (2017). Predicting the pathogenicity of novel variants in mitochondrial tRNA with MitoTIP. PLOS Comput Biol.

[42] Tafti M, Khatami M, Rezaei S, Heidari MM, Hadadzadeh M (2018). Novel and heteroplasmic mutations in mitochondrial tRNA genes in Brugada syndrome. Cardiol J.

[43] Timón-Gómez A, Nývltová E, Abriata LA, Vila AJ, Hosler J, Barrientos A (2018). Mitochondrial cytochrome C oxidase biogenesis: Recent developments. Semin Cell Dev Biol.

[44] Villar P, Bretón B, García-Pavía P, González-Páramos C, Blázquez A, Gómez-Bueno M, G (2013). Cardiac dysfunction in mitochondrial disease. Clinical and molecular features. Circ J.

[45] Wang L, Zhang Q, Yuan K, Yuan J (2021). mtDNA in the pathogenesis of cardiovascular diseases. Disease Markers.

[46] Wei R, Ni Y, Bazeley P, Grandhi S, Wang J, Li ST (2021). Mitochondrial DNA content is linked to cardiovascular disease patient phenotypes. J Am Heart Assoc.

[47] Wu W, He J, Shao X (2020). Incidence and mortality trend of congenital heart disease at the global, regional, and national level, 1990-2017. Medicine (Baltimore).

[48] Zhao D, Liu Y, Xu Z, Shen H, Chen S, Zhang S (2021). Integrative bioinformatics analysis revealed mitochondrial defects underlying hypoplastic left heart syndrome. Int J Gen Med.

[49] Zhou B, Tian R (2018). Mitochondrial dysfunction in pathophysiology of heart failure. J Clin Invest.

[50] Zhu HY, Wang SW, Martin LJ, Liu L, Li YH, Chen R (2009). The role of mitochondrial genome in essential hypertension in a Chinese Han population. Eur J Hum Genet.

